# Progress in Disease of Digestive Organs

**Published:** 1903-06-13

**Authors:** 


### Progress in Disease of Digestive Organs.

**Organic Acidity.**—M. I. Knapp [1] draws attention to some forms of gastritis due to the presence of moulds, Aspergilli and Penicillia, in the stomach which have been generally unnoticed. Macroscopically they appear as yellowish green or dark-red clusters, and under the microscope the fragments are often taken for bacilli. The patients complain of abnormal hunger, pains, and even cramps. The pain is relieved for a time by food, and may extend to the œsophagus, with thirst and heartburn and sometimes vomiting. Aspiration shows a large quantity of acid greenish fluid of strong odour and a bottom layer like fine flour. The quantity is due to spasm of the pylorus from irritation, and not to atony, the odour to succinic acid, the presence of which is shown by extraction with ether and by floating the clear extract on to a ferric chloride solution when a red-brown ring will be formed. The condition is favoured by mouldy foods, such as some cheeses, and by the amount of sugar in them. Lavage, mineral acids such as HCl at meals and a mixture of sulphuric and phosphoric acids 20 minutes after them will effect a cure. The food should be kept free from moulds. The disease is generally mistaken for excess of hydrochloric acid. Conditions due to the presence of active yeast in the stomach show great distension, a feeling of weight, and a bitter taste in the mouth. Acetic and butyric acids will be present and chains of yeast cells, not merely isolated ones. The treatment is much the same, but bread, tea, and coffee, and many green vegetables must be forbidden.

**Antiseptics** in atonic dyspepsia may be given in the form of a mixture of HCl., ginger, pure carbolic acid, and strychnia. This is strongly recommended by Guthrie Rankin.[2] For acid dyspepsia he advises bismuth carbonate 30 grains, carbolic acid 2 grains, sp. ammon. aromat. m. 30, liquor strychniæ m. 5 (though this involves the risk of the strychnia being precipitated), and, if there is much pain, liquor morphiæ m. 10. In nervous dyspepsia the main thing is to increase the quantity of food taken, and especially of easily assimilated fats. The stomach pain depends on the constitutional state and is not cured by local remedies, though antiseptics relieve the discomfort. Valerianate of zinc and arsenic are here indicated.

**Digestive Secretions.**—The work of Pawlow [3] has been described in recent numbers of this journal. We may here notice his proof that each kind of food stimulates the appropriate ferment, thus a bread diet increases the output of the amylolytic ferment of the pancreas. Again, he shows that food in the mouth, when an œsophageal fistula has been formed, causes a flow of gastric juice in the empty stomach, but if the vagi have been cut no flow takes place till the left vagus is stimulated. The vagus too is found to both stimulate and to inhibit the pancreatic functions. Mechanical stimuli to the stomach do not excite the flow of gastric juice, but psychic influences—*e.g.* the idea of food, do. Liebig's extract stimulates the gastric secretions, acids when they have passed from the stomach to the duodenum stimulate the pancreas, which is also affected by mental stimuli like the stomach. Sodium bicarbonate reduces the activity of gastric secretion in a marked degree. M. Einhorn [4] gives an elaborate discussion of the histology of the gastric mucous membrane found in stomach washings during various stomach disorders and concludes that the functional derangements of secretion are not caused by histological changes. The diagnosis of carcinoma may sometimes be made if a direct invasion of the gland substance by epithelial cells can be found, but in ordinary forms of dyspepsia the gland tissue is normal. Treatment therefore should be directed primarily to the general state of the body to improve the nutrition. However, in hyperchlorhydria a proteid diet is best, and in hypochlorhydria or achylia a carbohydrate one finely divided. Holsti [5] finds that morphia diminishes the amount of acid and the movements of the stomach in hyperchlorhydria, but the condition returns when it is left off. On the whole, belladonna seems to be preferable to morphia in the ordinary forms of dyspepsia unless the pain is very severe. There are two forms of diarrhœa in hyperchlorhydria according to Sunnont and Lerat.[6] The paroxysmal type appears in the night and after meals, and resembles the crises of Tabes. The chronic form shows morning evacuations and may be mistaken for colitis. It leads to great emaciation. and is difficult of cure. Lemonade and chlorides often give good results in these cases.

[1] Med. Rec., Sept. 9. [2] Med. Rec., Jan. 24. [3] Brit. Med. Jour., Jan. 17. [4] Amer. Jour. Med. Sci., Oct. [5] Brit. Med. Jour., Jan. 31. [6] Brit. Med. Jour., Feb. 21.

(*To be continued.*)

